# Comparative Proteomics of Ostreid Herpesvirus 1 and Pacific Oyster Interactions With Two Families Exhibiting Contrasted Susceptibility to Viral Infection

**DOI:** 10.3389/fimmu.2020.621994

**Published:** 2021-01-18

**Authors:** Maxime Leprêtre, Nicole Faury, Amélie Segarra, Stéphane Claverol, Lionel Degremont, Mélissa Palos-Ladeiro, Jean Armengaud, Tristan Renault, Benjamin Morga

**Affiliations:** ^1^ Université de Reims Champagne-Ardenne, UMR-I 02 INERIS-URCA-ULH SEBIO Unité Stress Environnementaux et BIOsurveillance des milieux aquatiques, UFR Sciences Exactes et Naturelles, Campus du Moulin de la Housse, Reims, France; ^2^ SG2M-LGPMM, Laboratoire De Génétique Et Pathologie Des Mollusques Marins, Ifremer, La Tremblade, France; ^3^ Department of Anatomy, Physiology & Cell Biology, School of Veterinary Medicine, University of California, Davis, CA, United States; ^4^ Centre Génomique Fonctionnelle de Bordeaux, Plateforme Protéome, Université de Bordeaux, Bordeaux, France; ^5^ Université Paris-Saclay, CEA, INRAE, DépartementMédicaments et Technologies pour la Santé (DMTS), SPI, Bagnols-sur-Cèze, France; ^6^ Département Ressources Biologiques Et Environnement, Ifremer, Nantes, France

**Keywords:** proteomics, interactions, antiviral response, OsHV-1, Pacific oysters

## Abstract

Massive mortality outbreaks affecting Pacific oysters (*Crassostrea gigas*) spat/juveniles are often associated with the detection of a herpesvirus called ostreid herpesvirus type 1 (OsHV-1). In this work, experimental infection trials of *C. gigas* spat with OsHV-1 were conducted using two contrasted Pacific oyster families for their susceptibility to viral infection. Live oysters were sampled at 12, 26, and 144 h post infection (hpi) to analyze host-pathogen interactions using comparative proteomics. Shotgun proteomics allowed the detection of seven viral proteins in infected oysters, some of them with potential immunomodulatoy functions. Viral proteins were mainly detected in susceptible oysters sampled at 26 hpi, which correlates with the mortality and viral load observed in this oyster family. Concerning the Pacific oyster proteome, more than 3,000 proteins were identified and contrasted proteomic responses were observed between infected A- and P-oysters, sampled at different post-injection times. Gene ontology (GO) and KEGG pathway enrichment analysis performed on significantly modulated proteins uncover the main immune processes (such as RNA interference, interferon-like pathway, antioxidant defense) which contribute to the defense and resistance of Pacific oysters to viral infection. In the more susceptible Pacific oysters, results suggest that OsHV-1 manipulate the molecular machinery of host immune response, in particular the autophagy system. This immunomodulation may lead to weakening and consecutively triggering death of Pacific oysters. The identification of several highly modulated and defense-related Pacific oyster proteins from the most resistant oysters supports the crucial role played by the innate immune system against OsHV-1 and the viral infection. Our results confirm the implication of proteins involved in an interferon-like pathway for efficient antiviral defenses and suggest that proteins involved in RNA interference process prevent viral replication in *C. gigas*. Overall, this study shows the interest of multi-omic approaches applied on groups of animals with differing sensitivities and provides novel insight into the interaction between Pacific oyster and OsHV-1 with key proteins involved in viral infection resistance.

## Introduction

Ostreid herpesvirus type 1 (OsHV-1) represents a major threat to the economy of the oyster aquaculture industry, as it is the causal agent of a severe disease that leads to massive oyster mortality and significant economic losses (1–3). Since 2008, a newly reported genotype of the virus was detected in association with mass mortality outbreaks of *Crassostrea gigas* first in France and then in other countries in Europe (4). Additional microvariants have been also reported since 2010 in New Zealand and Australia during mass mortality events affecting Pacific oysters (5–9). The implication of OsHV-1 in *C. gigas* mortalities has been proved in field condition (10) and by experimental infections performed by injection of viral suspension or cohabitation with experimentally infected animals (11, 12). The development of experimental infection methods opened new perspectives to study interactions between Pacific oysters and the virus OsHV-1.

Control of OsHV-1 infection is considered as a key element to maintain competitiveness and increase sustainability of the oyster industry. Pacific oysters, like other farmed marine bivalves, present unique challenges in terms of health management (13). Vaccination strategies that are currently used for other farmed animal species such as cattle and fish cannot be applied to Pacific oysters to prevent viral infections. However, the study of molecular mechanisms involved in viral-host interactions may give some information to limit the harmful effects of these pathogens. A significant genetic basis for resistance to OsHV-1 infection has been demonstrated in *C. gigas* (14, 15). Oyster families selected for their higher resistance or higher susceptibility to OsHV-1 have been developed and used broadly for other topics such as polyploids (16), breeding for OsHV-1 from different geographical origins (17), breeding for dual resistance (18, 19), or immunology (20). In this context, we investigated in this study the viral infection using two contrasted families (high versus low resistance) to explore the genetic basis of a better resistance to the viral infection and to better understand the molecular response of the host to the viral infection.

The molecular interactions between *C. gigas* and OsHV-1 have been investigated by transcriptomic analysis, using PCR-based approach or RNA sequencing technologies (20–22). These studies have led to the identification of key genes involved in host-viral interactions, such as genes of the antiviral defense. In contrast, few proteomic analyses have been conducted to investigate the molecular mechanisms involved in C. gigas infected with OsHV-1. To our knowledge, only Corporeau et al. (23) performed discovery proteomics analysis on Pacific oysters challenged with OsHv-1. Using two-dimensional electrophoresis, the authors observed a modulation of few proteins involved in cytoskeleton organization, stress response, signaling pathways and energy metabolism in oysters inoculated with OsHV-1 at high load compared to those inoculated at low load. Other proteomics analyses only focused on specific proteins of OsHV-1. Studies that used immunological tools to identify the tissue distribution of OsHV-1 in infected Pacific oysters have confirmed that viral proteins are mainly detectable in the connective tissues of different organs (24–26). Additionally, the detection of viral proteins was most often associated with histopathological changes previously reported by histology and transmission electron microscopy. Martenot et al. (25) showed that positive signals obtained by using three antibodies specific to viral proteins were at their maximal level within the initial 6 h after viral infection, and all studied organs appeared infected at 28 h post infection (hpi) demonstrating that the viral cycle begins quickly after experimental infection. Martenot et al. (27) showed that several viral proteins appeared to be involved in the entry of OsHV-1 into host cells.

By providing the quantification of thousands of proteins, shotgun proteomics is a promising tool to get insight into the molecular mechanisms of animals (28), and especially to decipher host-microbial interactions (29, 30). Indeed, proteomics has several advantages for assessing the effects of pathogens on the health of organisms. Proteins are the functional units of cells and their modulation are therefore more representative of phenotypes changes compared to gene expressions. Proteins can be regulated and modulated by post-translational reactions that cannot be investigated by transcriptomic or genomic approaches (31). This is especially true in oyster-OsHV-1 interactions. Indeed, a study has recently revealed that the translation of coding RNAs is highly regulated by non-coding micro-RNAs in naturally OsHV-1-infected oysters (32).

To investigate the molecular mechanisms behind a higher susceptibility or a better resistance to OsHV-1 infection, Segarra et al. (33) developed a study based on the induction of an experimental infection in Pacific oysters belonging to two families (named A and P) with contrasted susceptibility to OsHV-1. In the family A, the high mortality rates of infected oysters were correlated with a significant increase of viral DNA load, pointing to the sensitivity of the A-family to OsHV-1 (33). Conversely, infected oysters of the family P showed a lower mortality rate and a constant viral load in their tissues throughout the experiment, which could be explained by a better ability of P-oysters to fight the virus (33). Using qPCR technic, the authors also observed an increase of viral RNA in A-oysters over the time of infection and observed a dissimilar gene expression profiles between the two families, based on the modulation of a few antiviral genes over the time post-infection.

In this work, shotgun proteomics was applied on A- and P-oysters sampled from the experiment performed by Segarra et al. (33). By assessing, for the first time, the proteomic modulations of oysters with contrasted susceptibility to OsHV-1, this study aims to validate and complement previous transcriptomics studies to better characterize the molecular mechanisms underlying the differences in susceptibility to OsHV-1.

## Materials and Methods

### Experimental Infection of Pacific Oyster for Proteomics

The experimental infection of Pacific oysters was performed on two oyster families, produced from wild oysters sampled in Marennes Oléron Bay in January 2011 at the Ifremer hatchery in La Tremblade (France) (33). The production of these families has been described in Segarra et al. (33).

As described in Segarra et al. (33), 80 oysters per family (9 months old, 3 cm) were injected with 100 μl of viral suspension at 10^3^ copies of viral DNA/μl (μVar) into the adductor muscle. The same approach was performed for the control condition except that oysters were injected with artificial seawater. Then, oysters were distributed in 4 tanks per condition (control vs. infected) and per family (20 animals per tank). For each family and condition, one tank was dedicated to record survival and the three others were dedicated to the sampling for proteomics. For each tank, three live oysters were collected 12 and 26 h post-injection (hpi), as well 144 hpi for the family P. Whole oyster was flash frozen in liquid nitrogen and stored at −80°C. Three biological replicates per time point and family were selected for comparative proteomics.

### Protein Extraction

Recipient Pacific oyster was ground in liquid nitrogen in 50 ml stainless steel bowls with 20-mm-diameter grinding balls (Retsch MM400 mill). The powders obtained (stored at −80 °C) were then used for extract proteins. For this, 100 mg of powdered Pacific oysters were transferred into an Eppendorf tube 1.5 mL. One milliliter of extraction buffer (Trisma base, (ref T1503, SIGMA) 50 mM, EDTA (Ethylene Diamine Tetraacetic Acid, ref ED, SIGMA) 10 mM pH 8.3 and protease inhibitor mix (ref 80-6501-23, GE Healthcare) was added into the tube and mixed thoroughly. For each lysate, the total protein content was quantified using Bradford BCA-protein assay Kit (Prod #23227, ThermoScientific) with 96-well micro-plates (Nunc™) in a micro-plate reader (Multiskan EX Thermo) and Thermo labsystems ascent software to compare results with a calibration curve of Bovine Serum Abumin used as standard protein.

### Sample Preparation and Protein Digestion

Ten micrograms of each protein sample was solubilized in Laemmli buffer and were deposited onto SDS-PAGE gel 10% for concentration and cleaning purpose. Separation was stopped once proteins have fully entered the resolving gel. After colloidal blue staining, bands were cut out of the SDS-PAGE gel and subsequently cut in 1 mm x 1 mm gel pieces. Gel pieces were destained in 25 mM ammonium bicarbonate 50% acetonitrile (ACN), rinsed twice in ultrapure water and shrunk in ACN for 10 min. After ACN removal, gel pieces were dried at room temperature, covered with the trypsin solution (10 ng/µl in 50 mM NH_4_HCO_3_), rehydrated at 4°C for 10 min, and finally incubated overnight at 37°C. Samples were then incubated for 15 min in 50 mM NH_4_HCO_3_ at room temperature with rotary shaking. The supernatant was collected, and an H_2_O/ACN/HCOOH (47.5:47.5:5) extraction solution was added onto the gel slices for 15 min. The extraction step was repeated twice. Supernatants were pooled and dried in a vacuum. Pellets were finally resuspended in 100 µl of 0.1% formic acid and stored at -20°C.

### NanoLC-MS/MS analysis and Label-Free Quantitative Data Analysis

Peptide mixture was analyzed on a Ultimate 3000 nanoLC system (Dionex, Amsterdam, The Netherlands) coupled to an Electrospray Q-Exactive quadrupole Orbitrap benchtop mass spectrometer (Thermo Fisher Scientific, San Jose, CA). Ten microliters of peptide digests was loaded onto a 300-µm-inner diameter x 5 mm C18 PepMapTM trap column (LC Packings) at a flow rate of 30 µl/min. The peptides were eluted from the trap column onto an analytical 75 mm id x 25 cm C18 Pep-Map column (LC Packings) with a 4–40% linear gradient of solvent B in 108 min (solvent A was 0.1% formic acid in 5% ACN, and solvent B was 0.1% formic acid in 80% ACN). The separation flow rate was set at 300 nL/min. The mass spectrometer operated in positive ion mode at a 1.8-kV needle voltage. Data were acquired using Xcalibur 2.2 software in a data-dependent mode. MS scans (m/z 300–2,000) were recorded at a resolution of R = 70,000 (@ m/z 200) and an AGC target of 1 x 10^6^ ions collected within 100 ms. Dynamic exclusion was set to 30 s and top 15 ions were selected from fragmentation in HCD mode. MS/MS scans with a target value of 1 x 10^5^ ions were collected with a maximum fill time of 120 ms and a resolution of R = 35,000. Additionally, only +2 and +3 charged ions were selected for fragmentation. Other settings were as follows: heated capillary temperature, 260°C; normalized HCD collision energy of 25% and an isolation width of 3 m/z.

### Database Search and Results Processing

MS/MS spectra were searched by SEQUEST through Proteome Discoverer 2.4 (Thermo Fisher Scientific Inc.) against a database consisting of *Crassostrea gigas* entries from NCBI (41447 entries in March 2020) and 116 entries from the Pacific oyster herpesvirus (Uniprot 2020-01). Spectra from peptides higher than 5000 Da or lower than 350 Da were rejected. The search parameters were as follows: mass accuracy of the monoisotopic peptide precursor and peptide fragments was set to 10 ppm and 0.02 Da, respectively. Only b- and y-ions were considered for mass calculation. Oxidation of methionines (+16 Da) and protein N-terminal modifications (Acetylation +42 Da; Methionine loss -131 Da, Methionine-loss + Acetylation -89 Da) were considered as variable modifications and carbamidomethylation of cysteines (+57 Da) as fixed modification. Two missed trypsin cleavages were allowed. Peptide validation was performed using Percolator algorithm (34) and only “high confidence” peptides were retained corresponding to a 1% False Positive Rate (FDR) at peptide level. Peaks were detected and integrated using the Minora algorithm embedded in Proteome Discoverer. Proteins were quantified based on unique peptide intensities. Normalization was performed based on total protein amounts. Protein ratio was calculated as the median of all possible pairwise peptide ratios. A t-test was calculated based on the background population of peptides or proteins. Quantitative data were considered for proteins quantified by at least a unique peptide, fold changes (FC) above 1.5 and a statistical p-value lower than 0.05. For each family of oysters, FC were calculated by comparing the abundance of proteins between “infected” and their related “controls” conditions at all sampling times. Proteins were also considered as significantly modulated when at least two unique peptides were identified in one condition (i.e., infected) and not in the other (i.e., control). For these later, an arbitrary FC of 2 were assigned for bioinformatics analysis.

### Bioinformatic Analysis

To highlight the number of proteins that are specifically or commonly modulated between the different exposure conditions, differentially expressed proteins (DEPs) were displayed using a Venn diagram made with InteractiVenn (35).

To investigate the biological processes (BPs) involved in the virus-host interaction in both families, KEGG pathways and gene ontologies (GO) analyses were performed. The KO-Based Annotation System (KOBAS) was used to annotate the set of protein sequences with KO terms and identify enriched KEGG pathways in each exposure conditions (36, 37). The polypeptide sequences identified by LC-MS/MS were first assigned to KO terms using the *C. gigas* database of KOBAS. For enrichment analysis, DEPs were defined as input data and the whole set of proteins were used as a background. Still with KOBAS, Fisher’s exact tests were performed on DEPs in each condition. KEGG pathways were considered as enriched with a p-value lower than 0.05.

For GO annotation, the software Blast2go was used to search homologous annotations of the 3,204 polypeptide sequences identified by LC-MS/MS. First, a Blastp comparison against the Swissprot database was performed. Homologous annotations were validated with a maximum number of target hits of 20 and an E-value threshold set at 1E-03. From blast results, best hits were used to retrieve the GO-terms on uniport website, using Swissprot identities (IDs). Then, Go-terms related to BP categories were used as inputs for GO enrichment analysis. Enrichment analyses were performed as described in De Lorgeril and coworkers (20), with minor modifications for proteomic data. Briefly, analyses were done with an R and Perl script (available at “https://github.com/z0on/GO_MWU”) which combine a rank-based statistical test (Mann-Whitney U test) with an adaptive clustering of Go-term (the parameters were: largest 0.1, smallest 10, and clusterCutHeight 0.25). From the 3,207 proteins of *C. gigas* identified by LC-MS/MS, fold change values were assigned to the significantly modulated proteins (p-value < 0.05; FC > 2) and a “0” to the proteins that are not modulated in the tested condition. A GO category was considered as enriched with an adjusted p-value lower than 0.05 and an FDR lower than 10%. For both enriched KEGG pathways and GO-terms, a fold enrichment (FE) was estimated by dividing the number of significantly modulated proteins in a category by the total number of proteins assigned to the category and were expressed as a percentage. All results were summarized in graphics made with the R package ggplot2.

## Results

### Experimental Design and Acquisition of MS/MS Spectra

**Figure 1:**
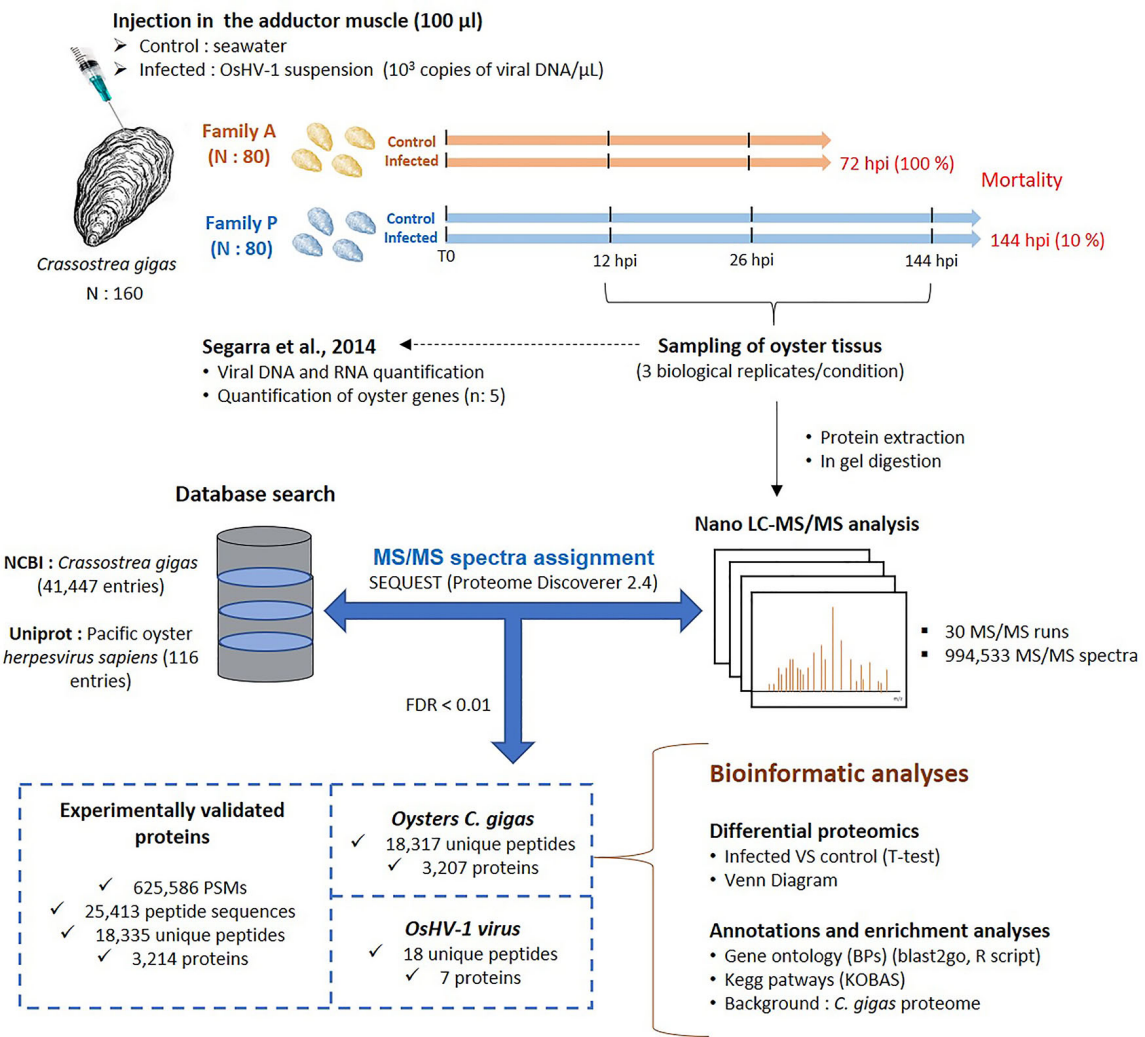
Experimental workflow and global view of key results of the experiment performed to investigate the proteomic response of oysters from families A and P challenged with the virus OsHV-1.

**Figure 1** describes the experimental design and MS/MS results. As reported by Seggara et al. (33), the mean survival rates of the infected oysters of the families A and P were 37% and 95% at 48 hpi, respectively, and 0% and 90% at 72 hpi. Beyond 72 hpi, the survival rate of the family P did not change with 89.5% at 144 hpi (**Figure 1**). For the control conditions, the survival rate was 100% for both families. Segarra et al. (33) assayed viral DNA by qPCR in the mantle of infected oysters to estimate the viral load over the time of infection. Their results showed a higher detection of viral DNA in mantle tissue sampled from the family A compared to the family P, with 1.5 and 3 log difference at 12 hpi and 26 hpi, respectively. For the family A, the amount of DNA copies jumped from 10^2^ at 12 hpi 10^7^ at 26 hpi. In contrast, the amount of DNA copies for the family P had a moderate increase from 10^1^ to 10^4^ but oysters displayed higher variability of viral content than at 12 hpi and 144 hpi. Finally, the amount of DNA copies of the P-oysters decreased to 10^2^ at 144 hpi.

With three biological replicates per condition and sampling times (12, 26, and 144 hpi), a total of 30 runs of LC-MS/MS were performed after protein extraction and trypsin digestion (**Figure 1**). A total of 994,533 MS/MS spectra were recorded and assigned using the NCBI database of *C. gigas* (41,447 polypeptide sequences) and the uniprot database of OsHV-1 (116 polypeptide sequences) (data in **Supplementary File S1**). From the whole dataset, 38% of MS/MS spectra could be interpreted in terms of peptide sequences, a ratio comparable to other studies on aquatic animals (38). The resulting 625,586 peptide-spectrum matches (PSMs) pointed at the identification of 25,413 peptide sequences and the qualification of 3,214 proteins (**Figure 1**). Among them, 18,317 unique peptides were related to the *C. gigas* proteome, resulting in the identification of 3,207 oyster proteins (**Figure 1**). For OsHV-1, only 18 unique peptides were identified, and 7 proteins were validated (**Figure 1**).

### Detection of Viral Proteins in Infected Oysters

Among the 7 viral proteins detected in oysters challenged with OsHV-1, 6 were detected in the family A, while only 3 viral proteins were detected in the P-oysters (**Table 1**). The ORF 107 was exclusively detected in A-oysters sampled at 12 hpi. Most of the viral proteins (n: 5) were detected A-oysters sampled at 26 hpi. Among these proteins, two of them (ORF 27 and 75) contained a deoxyuridine triphosphate nucleotidohydrolase (dUTPase) domain while the ORF 104 and the ORF 45 contained a DNA translocase and a signal recognition particle (SRP) domains, respectively. In the family P, the ORF 27 was exclusively detected in oysters sampled at 12 hpi, while the ORF 82 and the ORF 104 were exclusively detected at 26 and 144 hpi, respectively.

**Table 1:** Viral proteins detected in A- and P- oysters challenged with OsHV-1.

Viral proteins		Detection in A-oysters		Detection in P-oysters		
Proteins	Conserved domains	12 hpi	26 hpi	12 hpi	26 hpi	144 hpi
ORF 104	DNA translocase FtsK		X			X
ORF 107	–	X				
ORF 27	dUTPase		X	X		
ORF 90	–		X			
ORF 75	dUTPase		X			
ORF 82	–				X	
ORF 45	SRP-docking protein FtsY		X			

### Modulation of Oyster Proteomes

**Figure 2:**
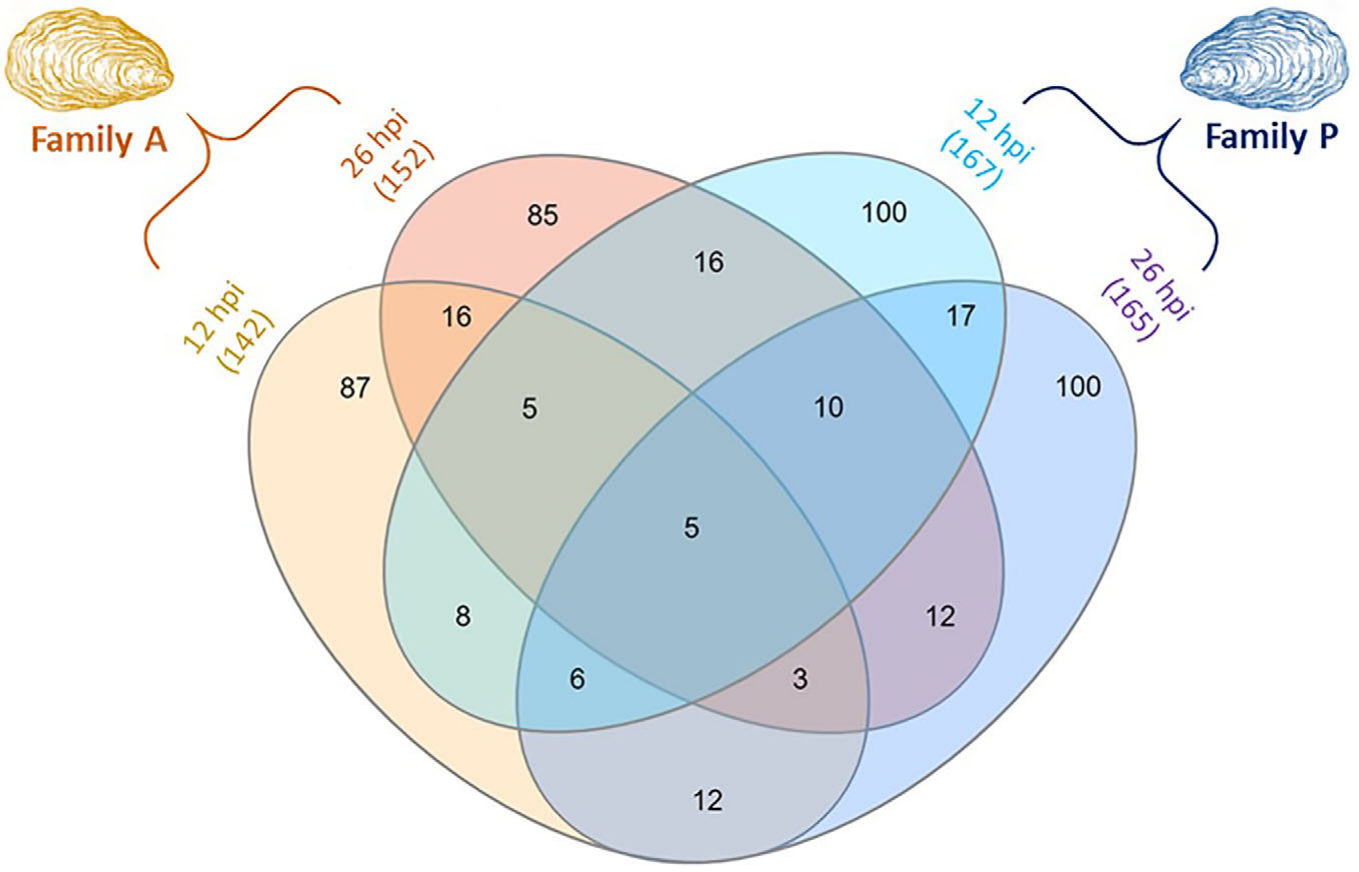
Venn diagram displaying the number of differentially expressed proteins (DEPs) in challenged Pacific oysters of the two families A (susceptible to OsHV-1) and P (more resistant to OsHV-1), sampled at 12 and 26 hpi. The numbers in brackets represent the total number of DEPs per condition. The number of DEPs in P-oysters sampled at 144 hpi is not presented in this figure.

For both Pacific oyster families and for each sampling time, a t-test was used to highlight significantly modulated protein abundance in A- and P-oysters exposed to OsHV-1 in comparison to the control condition. Protein abundance was considered as significantly modulated with an adjusted p-value lower than 0.05 and a fold change greater than 1.5. A total of 142 and 152 proteins were significantly modulated in infected Pacific oysters of the family A at 12 and 26 hpi, respectively (**Figure 2**). In P-oysters challenged with OsHV-1, the abundance of 167 and 165 proteins was modulated at 12 hpi and 26 hpi, respectively (**Figure 2**). A lower modulation was observed for the P-oysters at 144 hpi, since only the abundance of 119 proteins was significantly modulated in infected Pacific oysters (**Supplementary File S1**).

The number of differentially expressed proteins (DEPs) was displayed using a Venn diagram to address common or specific responses between A- and P-oysters exposed to OsHV-1 after 12 and 26 hpi (**Figure 2**). The Venn diagram shows distinct patterns of modulation between each exposure condition. Only five proteins were common between the four exposure conditions. These proteins were mainly related to the signal transduction and the immune defense, such as a G-coupled receptor and natterin-like proteins (**Supplementary Data S1**). In each condition, more than 50% of DEPs were exclusive to the experimental treatment. For the family A, 87 proteins and 85 proteins were exclusively modulated at 12 hpi and 26 hpi, respectively. In the family P, 100 proteins were exclusively modulated at 12 and 26 hpi. Few DEPs were shared between the two oyster families (**Figure 2**). Only 24 DEPs were common to the Pacific oyster families A and P at 12 hpi and the detection of 30 proteins was modulated in both A and P Pacific oysters sampled at 26 hpi. In addition, different patterns of modulations were also observed over time of exposure to OsHV-1 in each Pacific oyster family. For oysters belonging to the family A, the expression of 29 proteins was modulated in animals sampled at 12 and 26 hpi. Twice as much DEPs were common between P-oysters sampled at 12 and 26 hpi, since 48 DEPs were in common. For oysters belonging to the P family, a specific pattern of modulation was observed at 144 hpi (data not shown). Among the 119 DEPs, 65 were exclusive to this condition, the abundance of 26 and 21 DEPs was also modulated in A-oysters sampled at 12 hpi and P-oysters sampled at 26 hpi, respectively (**Supplementary File S1**).

### Enriched Biological Processes and KEGG Pathways

#### Gene Ontology Analysis

**Figure 3:**
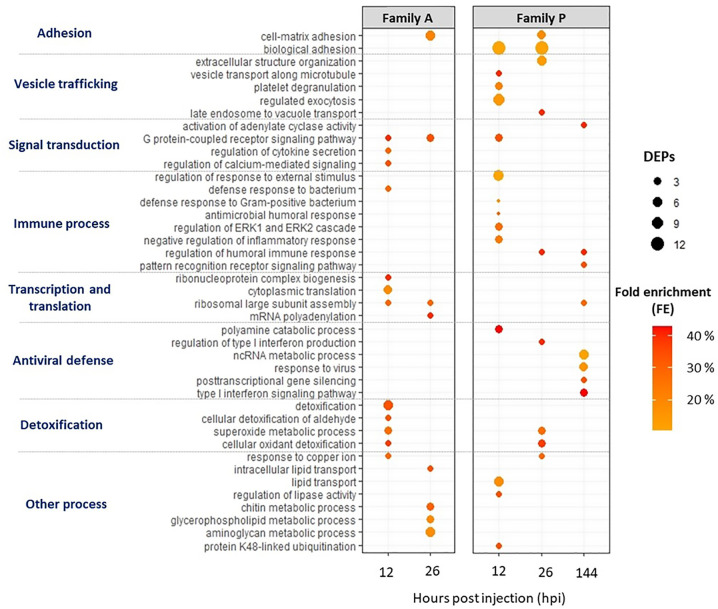
Biological process (BPs) Go-terms enriched for infected A- (susceptible to OsHV-1) and P-oysters (more resistant to OsHV-1) sampled at 12, 26 and 144 h post injection (hpi). The Go analysis was performed on DEPs using a rank-based statistical test (Mann-Whitney U test) and BPs were considered as enriched with an FDR lower than 10%. The number of DEPs assigned to BPs are represented by the bubble, and fold enrichments (FEs) are indicated by the intensity of the bubbles’ coloring.

GO IDs based on Swissprot annotations were used to assign and classify *C. gigas* proteins with Go-terms (BPs). Overall, 2,675 proteins matched with at least one BP GO-term, which represents more than 80% of the Pacific oyster proteome. DEPs were then subjected to a rank-based gene ontology analysis to evaluate the BPs in A- and P-oysters sampled at 12, 26, and 144 hpi. Results of the Go analysis are summarized in **Figure 3**.

Several BPs were enriched in both A- and P-oysters exposed to OsHV-1 (**Figure 3**). BPs related to cell adhesion were enriched at 26 hpi for Pacific oysters belonging to the family A and 12 and 26 hpi for animals belonging to the family P, with a higher number of DEPs compared to A-oysters. Signal transduction and detoxification-related BPs were also enriched in Pacific oysters belonging to both families. Among those involved in signal transduction, “G protein-coupled receptor signaling pathway” and “activation of adenylate cyclase activity” were enriched with a high FE in A-oysters sampled at 12 and 26 hpi and P-oysters sampled at 12 and 144 hpi. BPs related to detoxification processes were enriched earlier in Pacific oysters of the family A (12 hpi) compared to the family P (26 hpi) (**Figure 3**). These BPs included “superoxide metabolic process” and “cellular detoxification” in both A- and P- oysters, represented by the modulation of antioxidant enzymes such as the superoxide dismutases (SOD) or the glutathione S-transferases (GSTs), modulated in individuals belonging to both Pacific oyster families (**Table 2**). However, “Detoxification” and “cellular detoxification of aldehydes” BPs were enriched exclusively in A-oysters sampled at 12 h. Many pattern recognition receptors (PRRs) were also modulated in individuals belonging to both families of Pacific oysters with different patterns of modulation (**Table 2**). Of them, a beta-1,3-glucan-binding protein (BGBP) and a hepatic lectin were positively modulated in A-oysters sampled at 26 hpi and negatively modulated in P-oysters sampled at 12 and 26 hpi. A C1q tumor necrosis factor (C1q-TNF) was exclusively modulated in A-oysters, while C1q, chitinase, and DM9-containg proteins were exclusively modulated in P-oysters.

**Table 2:** Example of differentially expressed proteins (DEPs) for A- (susceptible to OsHV-1) and P-oysters (more resistant to OsHV-1) over times post infection.

	Family A		Family P		
	12 hpi	26 hpi	12 hpi	26 hpi	144 hpi
**Pattern recognition receptors (PRRs)**					
C1q tumor necrosis factor-related protein [C1q-TNF]	4.4	-2.2	–	–	–
beta-1,3-glucan-binding protein [BGBP]	–	2.3	-3.9	-2.5	–
hepatic lectin	–	2.5	-4.4	-2.3	–
C1q-like protein [C1q]	–	–	-4.8	-4.1	–
DM9-containing protein [DM9CP]	–	–	–	1.8	–
chitinase 3	–	–	–	3.8	–
**Signal transduction**					
**Immune signaling**					
LAMTOR1-like protein	2.8	–	–	–	–
LRR-containing G-protein coupled receptor	6.5	-2.0	-2.3	–	–
Signal transducer and activator of transcription 5A-like [STAT]	–	-2.2	–	–	–
Interferon-induced protein 44-like [IFI44]	–	–	1.8	–	3.0
**Cytokines**					
Allograft inflammatory factor 1-like [AIF-1]	–	2.1	–	–	–
Tumor necrosis factor ligand [TNF-α]	–	-3.5	–	–	–
**Ubiquitination**					
Ubiquitin-conjugating enzyme E2	–	–	2.4	–	–
Ubiquitin-like-specific protease ESD4	–	–	2.4	2.1	–
E3 ubiquitin-protein ligase UBR5	–	–	–	2.1	–
Ubiquitin protein	–	–	–	3.0	2.4
**Immune effectors**					
**Lysosomal protein**					
V-type proton ATPase	–	3.1	–	–	–
lysosomal aspartic protease	–	2.3	–	–	–
cathepsin L1 isoform X2	–	3.7	–	–	–
cathepsin B	–	2.1	–	–	–
cathepsin L	–	3.6	–	-2.6	–
**mRNA surveillance**					
Diamine acetyltransferase (SSAT-1)	-3.3	–	1.8	–	–
mRNA-decapping enzyme	–	–	2.9	–	–
U6 snRNA-associated Sm-like protein LSm8	–	–	2.8	2.1	–
Tetratricopeptide repeat protein	–	–	–	2.7	–
**RNA interference, RIG-I like pathway**					
Serrate RNA effector	–	–	1.9	–	2.2
Protein argonaute-2	–	–	–	–	2.5
interferon-induced helicase C	–	–	–	2.0	2.0
**Other immune effectors**			–	–	–
laccase-24	–	–	–	2.6	–
**Antioxydant metabolism**					
Superoxide dismutase [Cu-Zn]	3.2	–	–	–	–
Superoxide dismutase [Cu-Zn]-like precursor	2.2	–	–	2.3	–
Glutathione S-transferase Mu 3	–	–	3.2	12.3	–
Cavortin	–	–	-2.6	–	–

Proteins were considered as DEPs with a p-value < 0.05 and a fold change (infected vs control) higher than 2. Positive FCs are represented in green and negative FCs are represented in red.

Most of enriched BPs differed between individuals belonging to the two families of Pacific oysters challenged with the virus. In A-oysters sampled at 12 hpi, the BPs “cytokine secretion”, “calcium signaling” and “defense response to bacterium” were each enriched with a FE greater than 30% and 3 DEPs (**Figure 3**). These BPs included the modulation of two cytokines named allograft inflammatory factor (AIF-1) and tumor necrosis factor ligand (TNF) (**Table 2**). At both 12 and 26 hpi, several BPs involved in the ribosome biogenesis as well as the transcription and translation of the DNA/RNA were enriched in A-oysters challenged with OsHV-1 (**Figure 3**). Other BPs related to the “response to copper ions” and “lipid metabolic processes” were enriched in A-oysters sampled at 12 and 26 hpi, respectively.

As for Pacific oysters belonging to the A-family, several BPs were exclusively enriched in infected oysters of the family P. BPs that involve vesicular trafficking, were enriched at 12 and 26 hpi and included “regulated exocytosis” and “extracellular structure organization” BPs (**Figure 3**). Among them, “vesicle transport” and “late endosome to vacuole transport” were the most enriched BPs (FE > 40%) at 12 and 26 hpi, respectively. Immune-related BPs were mainly enriched in P-oysters and were different over the time post infection. Indeed, BPs related to the “antimicrobial defense” and “inflammatory response” were exclusively enriched in infected P-oysters sampled at 12 hpi. The BP “regulation of humoral responses” was enriched (FE > 40%) in infected P-oysters sampled at 26 and 144 hpi, while the BP “pattern recognition signaling pathway” was only enriched at 144 hpi with few DEPs (**Figure 3**).

As expected, several BPs specific to the antiviral response were enriched in infected P-oysters (**Figure 3**). The BP “polyamine catabolic process” BP was enriched at 12 hpi with three diamine acetyltransferases (SSAT). Four BPs involved in the antiviral defense were enriched in P-oysters sampled at 144 hpi. From these BPs, “type 1 interferon signaling pathway” BP was the most enriched (FE > 40%), followed by the BPs “response to virus” and “ncRNA catabolic process”, both enriched with more than five proteins. Then, the BPs related to “gene silencing” were enriched with a FE greater than 30%. BPs related to the antiviral defense were supported by the positive modulation of numerous potential antiviral proteins in P-oysters. Among them, a serrate RNA effector was modulated at 12 and 144 hpi, the interferon-induced helicase C was modulated at both 26 and 144 hpi, and many isoform of interferon-induced protein 44-like (IFI44) were modulated in infected P-oysters sampled at 12 and 144 hpi (**Table 2**). Finally, a BP linked to “ubiquitination process” was enriched in P-oysters sampled at 12 hpi and several ubiquitin-related proteins were modulated in P-oysters, throughout the course of infection (**Figure 3**, **Table 2**).

#### KEGG Pathway Analysis

**Figure 4:**
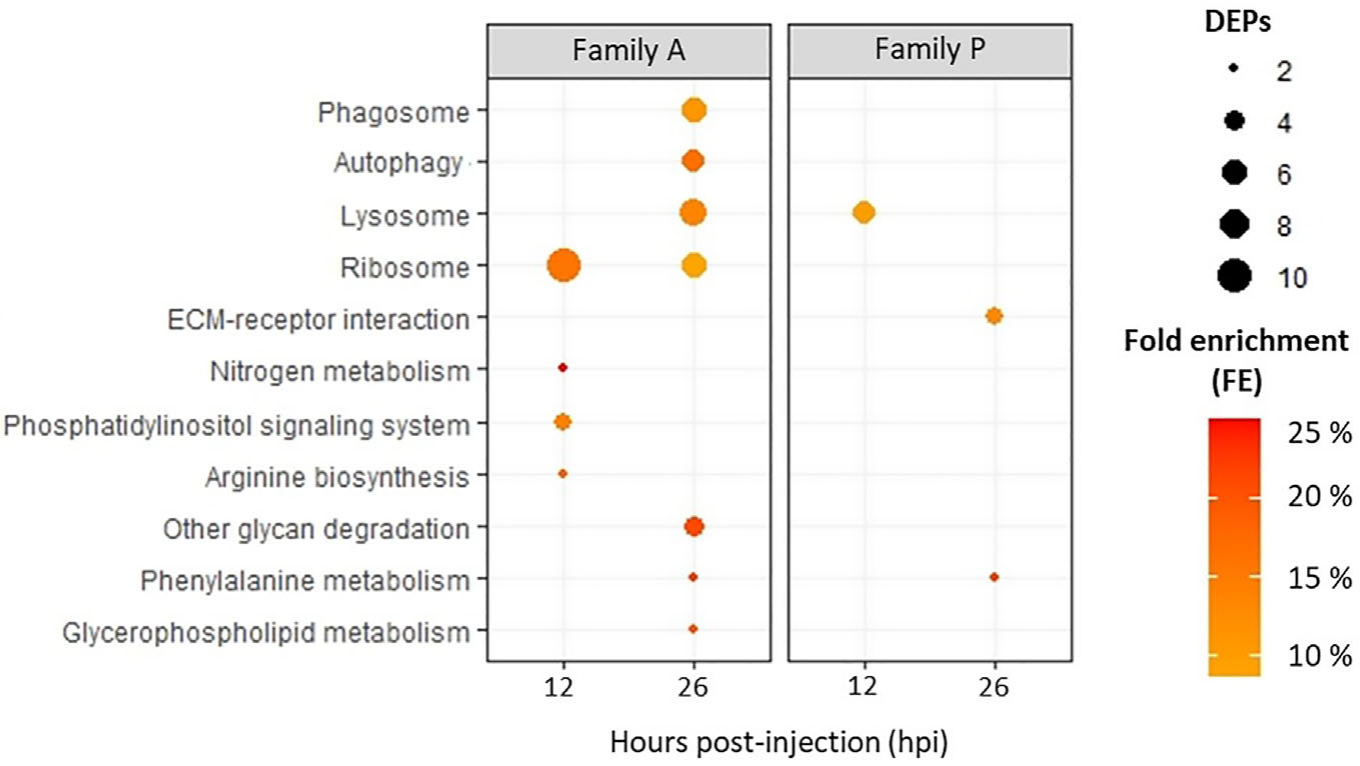
KEGG pathways enriched for Pacific oysters of the family A and P, challenged with OsHV-1 during 12 and 26 hpi. According to DEPs, KEGG pathways were considered as enriched according to a Fischer exact test (p-value < 0.05). The number of DEPs assigned to KEGG pathways are represented by the bubble, and fold enrichments (FEs) are indicated by the intensity of the bubbles’ coloring.

Using the annotation tool of KOBAS against *C. gigas* database, 1,096 proteins could be assigned to at least one KEGG pathway, representing about 35% of the 3,207 proteins identified. Considering all exposure conditions, only 10 KEGG pathways were enriched (**Figure 4**). A greater number of pathways were enriched in Pacific oysters from the family A compared to oysters from the family P. Four KEGG pathways were enriched in A-oysters sampled at 12 hpi. These included the KEGG pathways “nitrogen metabolism” and “arginine biosynthesis”, both enriched with two proteins, and the pathway “Phosphatidylinositol signaling system”, enriched with three proteins. The pathway “Ribosome” was enriched in Pacific oysters of the family A sampled at 12 hpi (11 proteins) and 26 hpi (6 proteins). Still in the family A, more KEGG pathways were enriched in the Pacific oysters sampled at 26 hpi (7 pathways) compared to those sampled at 12 hpi (four pathways). Many of them were related to the endocytosis processes of micro-organisms. Indeed, “lysosome”, “phagosome”, and “autophagy” pathways were enriched with more than five proteins (**Figure 4**). These pathways were linked to the positive modulation of numerous lysosomal proteins, such as cathepsins which were modulated with fold changes greater than 3 (**Table 2**). Other pathways such as the “glycan degradation”, “glycerol phospholipid and phenylalanine metabolisms” were also enriched in infected A-oysters sampled at 26 hpi. Regarding the Pacific oysters of the family P, only three KEGG pathways were enriched at 12 and 26 hpi. Like the A-oysters sampled at 26 hpi, the “lysosome” pathway was enriched in P-oysters sampled at 12 hpi with five proteins. For P-oysters sampled at 26 hpi, “phenylalanine metabolism” and the ”extracellular matrix (EMC) receptor interaction” KEGG pathways were enriched with two and three proteins, respectively. No KEGG pathways were enriched for the P-oysters sampled infected during 144 hpi.

## Discussion

Shotgun proteomics was applied to investigate the molecular interactions between Pacific oysters and the virus OsHV-1. For this purpose, proteomic modulations of two families of oysters with contrasted susceptibility to OsHV-1 were studied following an experimental infection (33). To infect oysters, authors performed experimental injections of OsHV-1 in the adductor muscle of Pacific oysters, an experimental approach developed by Schikorski et al. (11) which is routinely used to evaluate *in vivo* interactions between *C. gigas* and OsHV-1 (33, 39–41). Infection by injection provides a better synchronization of the viral infection compared to natural infections. Indeed, by introducing the same amount of viral load into the oysters, injections seem more suitable to compare the biological responses occurring in the tissues of oysters challenged with OsHV-1. However, it is important to remember that this methodology does not consider the natural entry of the virus into Pacific oysters. In a natural infection, viruses face physical barriers and mucosal immune responses of oysters, which are overlooked when infections are performed by injection (42). The increase of the viral DNA and RNA load as well as the high mortality rate observed in A-oysters injected with OsHV-1, over the time of infection, indicated the success of the experimental infection as well as the high susceptibility of A-oysters to OsHV-1. In contrast, the oysters belonging to the family P were considered as resistant to OsHV-1, since low viral loads were quantified in their tissues throughout the experiment and the mortality rate of infected P-oysters were lower than 10% at 144 hpi. These contrasting phenotypic traits make both families of interest for evaluating the molecular mechanisms of susceptibility and resistance of Pacific oysters facing the virus.

While 39 viral genes were assayed by qPCR in Segarra et al. (33), only 7 viral proteins were detected in oysters challenged with OsHV-1. In shotgun proteomics, only the most intense ions are analyzed by the mass spectrometer, which limits the detection of low abundance peptide ions and thus the identification of low abundance proteins (43). In this case, we can suspect that these viral proteins were present in large quantities compared to other viral proteins. More viral proteins were detected in the infected oysters belonging to the family A compared to the family P, confirming the high replication of the virus in the A-oysters. Among them, two dUTPase-like proteins (ORF 27 and 75) were also highly expressed at the transcriptomic level in Segarra et al. (33), suggesting that they are produced in high abundance. dUTPases from herpes viruses were reviewed by Williams et al. (44). Authors described the dUTPases as key modulators of the host immune responses that can alter the inflammatory microenvironment of human diseases. Considering their potential immunomodulatory functions, these two enzymes from OsHV-1 may perturb the immune response of Pacific oysters. The ORF 104, which contains a DNA translocase domain, was detected after 26 hpi in A-oysters and after 144 hpi in P-oysters. The ORF 82 was also detected after 26 hpi, but only in oysters belonging to the family P. Our proteomic results correlate with the viral genes expression reported by Morga et al. (45). Indeed, the authors showed that the genes coding for these two ORFs were highly expressed after 24 h in the hemocytes of *C. gigas* exposed *in vitro* to OshV-1.

From the 30 biological samples analyzed by LC-MS/MS, more than 3,000 oyster proteins could be identified and quantified, a result similar to other recent proteomic analyses performed on bivalves (46, 47). Our proteomic analysis was performed on the whole tissues of Pacific oysters to assess the general response of infected animals. Thus, the whole proteome of Pacific oysters was analyzed, which is not favorable to the quantification of low abundance proteins. Indeed, proteomics has a lower depth of analysis than transcriptomics approaches, since abundant proteins can mask the signal of low abundant proteins. However, protein modulations are more representative of phenotypic variations than changes in gene expression, which does not necessarily lead to protein synthesis, and altered phenotype. Thus, our analysis complements previous transcriptomic studies that aimed to elucidate the molecular mechanisms involved in the antiviral defense of Pacific oysters during OsHV-1 infection (33, 39).

### Common Response to OsHV-1

While most of DEPs differed between both Pacific oyster families, some were modulated in both Pacific oyster groups upon infection. Among them, proteins involved in the G protein-coupled receptors (GPCRs) signaling pathway were modulated throughout the course of infection. GPCRs are regulators of signal transduction and are considered as important mediators of the immune defense in invertebrates (48). GPCRs also appear to play an important role in the antiviral response of oysters (He et al., 2015). Indeed, the authors reported a high expression of GPCR-related genes in the gills of *C. gigas* oysters infected with OsHV-1, highlighting their role in the signal transduction during host-viral interactions. Numerous proteins involved in the antioxidant defense were also modulated in both families of Pacific oysters but at different time points post infection. Antioxidant enzymes, such as SODs, were modulated earlier (12 hpi) in A-oysters infected with OsHV-1 compared to P-oysters (26 hpi). By removing free radicals, antioxidant enzymes are generally linked to the general stress response of bivalves (49). In *oysters* and other mollusks, antioxidant enzymes such as SODs, GSTs, and catalase are also considered as powerful immune effectors, acting on the elimination of microorganisms (50–53). Many studies have reported changes in gene expression and activity of antioxidant enzymes in Pacific oysters infected with pathogens, including OsHV-1 (20, 23, 54). In our case, the modulation of antioxidant proteins may reflect an earlier cellular stress and/or immune responses in oysters of the family A infected with OsHV-1, which is consistent with viral DNA loads observed in their mantle.

### Deficient Response in More Susceptible Pacific Oysters

**Figure 5:**
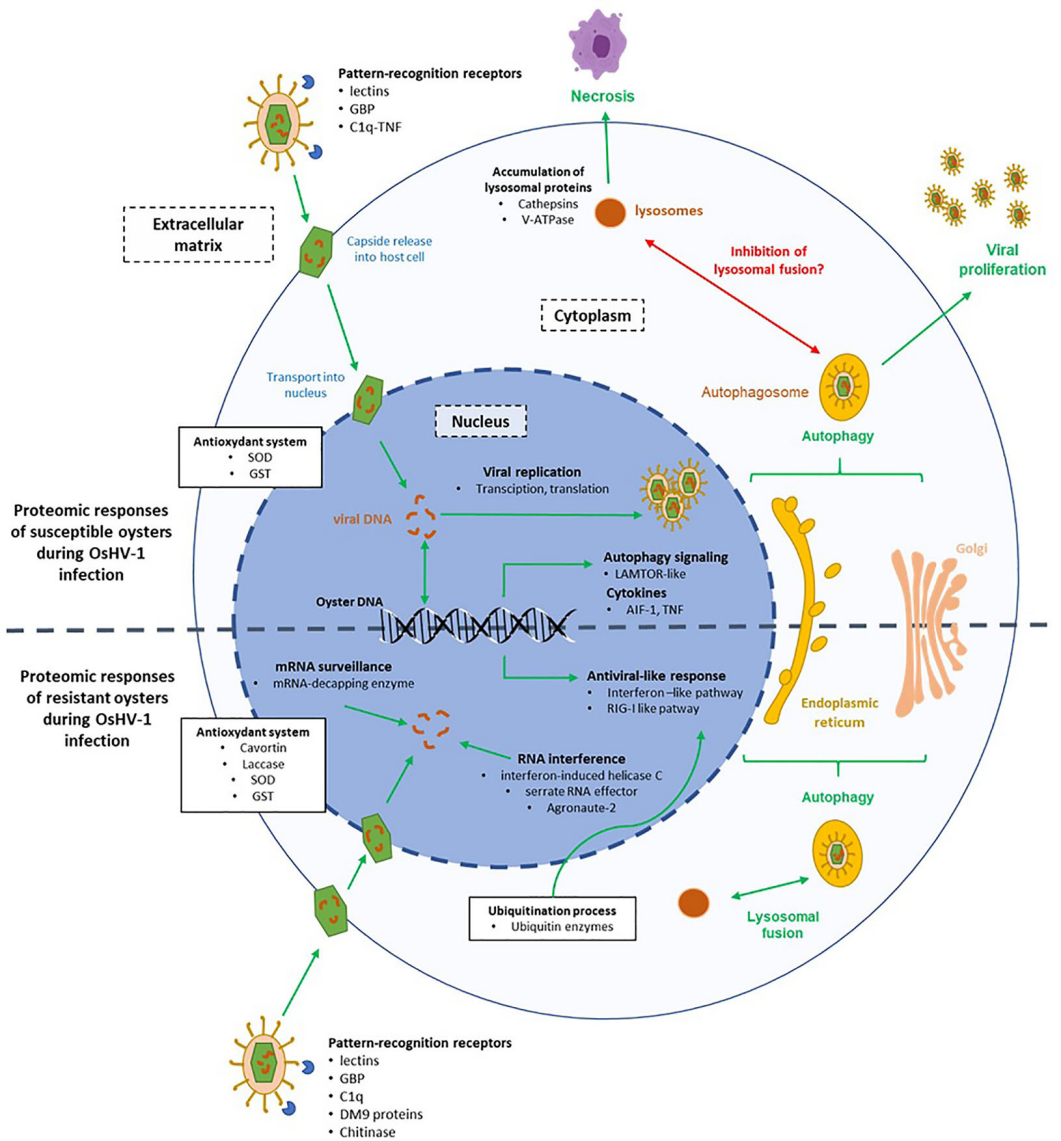
Hypothesis on the molecular mechanisms involved in the interaction between the virus OsHV-1 and cells of susceptible (A-family) and resistant (P-family) oysters. The green arrows represent pathways involved in response to the virus infection and the red arrows represent pathways inhibited by the virus.

Numerous proteins involved in DNA transcription and mRNA translation, such as helicases or ribosomes, were modulated in A-oysters infected with OsHV-1 (**Figure 5**). Like other viruses, herpesviruses hijack the host molecular machinery to replicate their DNA and proliferate in host cells (55). Given the high viral DNA load and high mortality rates observed in infected A-oysters (33), these results reinforce the idea that the virus readily proliferates in this Pacific oyster family.

Several clues indicated that autophagy is occurring in cells of infected A-oysters (**Figure 5**). Many KEGG pathways related to endocytosis and exocytosis processes, including “autophagy” and “lysosome” pathways, were enriched in Pacific oysters sampled at 26 hpi. These results were confirmed by the modulation of LAMTOR-1, a regulator of lysosomes and autophagosomes biogenesis (56). Moreover, many DEPs in A-oysters were linked to the calcium-mediated signaling pathway, which is known to influence the induction of autophagy (57, 58). The induction of the autophagy pathway in OsHV-1-infected Pacific oysters has been proven using molecular and cellular approaches (59–61). Autophagy is a major degradation system that protects animals from microorganism infections, including viruses. This process involves the delivery of cytoplasmic material to lysosomes *via* the autophagosome for the destruction of microorganisms (62). The role of autophagy in the elimination of herpesviruses has been highlighted by some studies (63, 64). It is also proven that herpesviruses can hijack autophagy to evade the host immune response and optimize their propagation or persistence (65). Some of them block the maturation of autophagosomes (lysosomal fusion) to avoid their degradation by lysosomal proteins while others can stimulate the whole process of autophagy for their transport in the cytoplasm (64). In our case, the high abundance of lysosomal proteins in A-oysters sampled at 26 hpi may reflect an accumulation of lysosomal content triggered by the induction of autophagy mechanisms. Moreau et al. (59) showed that the autophagy process was modulated differently depending on Pacific oyster family lines, suggesting a genetic basis in the fight against viral infection through autophagy. OsHV-1 could block the fusion of the autophagosome with the lysosome to escape oyster immune response, leading to an accumulation of lysosomal proteins in cells (**Figure 5**). This result is in accordance with the hypothesis proposed by Moreau et al. (59), based on the observed accumulation of the autophagy-related protein LC3-II in infected Pacific oysters after 24 hpi.

Lysosomal proteins, in particular cathepsins, are also considered as important regulators of cell death and inflammatory responses (66, 67). The involvement of cathepsins in virus-induced cell death has been highlighted by several studies, including in the context of herpesvirus infections (68–70). Thus, the high accumulation of cathepsins in A-oysters infected with OsHV-1 may result in the induction of cell death and/or inflammatory reactions (**Figure 5**). This latter hypothesis was supported by the modulation of the pro-inflammatory cytokine AIF-1, considered as key regulator of inflammation and cell death in mollusks, such as bivalve species (71, 72). Many clues indicate that apoptosis is inhibited when oysters are infected by OsHV-1. Indeed, the OsHV-1 genome codes for several anti-apoptotic proteins (27) and several anti-apoptotic genes are expressed in oysters challenged with OsHV-1, including the oysters from the family A (33, 73). Using molecular and cellular technics, Martenot et al. (73) observed an increase in cell death and a decrease of apoptosis processes in hemocytes sampled from infected oysters. In our study, no modulations of apoptotic effectors were observed, and the down modulation of a TNF-related protein in A-oyster suggests that apoptosis does not occur in the A-oysters challenged with OsHV-1 (73). Such result indicates that cell death of infected oysters is more linked to necrosis than apoptosis, certainly mediated by the increase of lysosomal proteins in oyster cells (**Figure 5**).

Overall, a significant modulation of immune-related proteins was observed in the more susceptible Pacific oysters to OsHV-1. The high accumulation of lysosomal proteins and/or a disorder in immune response may result in disease and related mortality. In other words, in the absence of feedback control, the enhanced production of defense-related proteins and/or the stimulation of defense mechanisms could become harmful for the host by causing tissue damage. As hypothesized by Segarra et al. (33), these Pacific oysters do not have an appropriate immune response to stop the OsHV-1 infection. We suspect that the virus is able to manipulate the antiviral response of *C. gigas*. For example, this study suggests that the virus is capable to highjack the autophagy pathway by blocking the fusion of autophagosomes with lysosomes in Pacific oysters belonging to Family A. Moreover, classical pathways of the antiviral response of *C. gigas* were not modulated at all, resulting in the proliferation of OsHV-1 in oyster tissues. Therefore, Pacific oysters were unable to fight the virus, and the increase of the viral load resulted in cell death and oyster mortality.

### Effective Antiviral Response in More Resistant Pacific Oysters

The proteomic responses observed in infected P-oysters indicated an appropriate and effective antiviral response against OsHV-1 allowing full control of the viral infection (**Figure 5**). A contrasted modulation of PRRs was observed between A- and P-oysters. Some of them were modulated in both families (BGBP and lectins) but earlier in infected P-oysters compared to A-oysters. Indeed, these PRRs were modulated in the first hours of the infection (12 hpi) in the family P, while those were modulated at 26 hpi in the family A, when the amount of viral load in oyster tissues was already high (33). Others were exclusively modulated in A-oysters (C1q-TNF) or P-oysters (C1q and a chitinase). Recognition of viral particles by PRRs is a critical step to initiate an effective immune response against microorganisms, including viruses (74, 75). In addition, research on mollusks showed that PRRs play a major role in the resistance to various pathogens (76–78). In the context of OsHV-1 infection of Pacific oysters, De Lorgeril et al. (79) observed different basal expressions of PRR genes in Pacific oyster families with contrasted susceptibilities to OsHV-1 infection. In our study, the contrasted modulation patterns of PRRs observed in A and P-oysters underline the crucial role of PRRs in the oyster’s resistance to OsHV-1 from the first hours of infection. The efficient recognition of the viral particles in P-oysters may trigger the activation of signaling pathways, leading to the production of appropriate antiviral responses.

In P-oysters infected with OsHV-1, a significant modulation of the ubiquitin-like proteins was observed (**Figure 5**). The ubiquitin system is considered as an important regulator of the innate sensing pathways initiated by PRRs. In the context of viral infection, ubiquitin-related proteins coordinate an effective antiviral immune response by regulating efficient antiviral signaling pathways such as the RIG-I-like, Toll-like or interferon-like pathways (80). Several studies showed that genes related to the ubiquitination process increased during the first hours of infection (6 and 12 hpi) with a higher expression in more resistant compared to more susceptible Pacific oysters (20, 41, 79). In our study ubiquitin-related proteins were highly modulated in the more resistant Pacific oysters, while few of them were modulated in the more susceptible Pacific oysters. In complement to previous studies, this differential proteomic analysis is in accordance with a critical role of the ubiquitin system in the defense against OsHV-1 infection. As shown for certain herpesviruses, it is possible that OsHV-1 manipulates the ubiquitin system of the more susceptible Pacific oysters to escape the antiviral defense and favor their proliferation in host cells (81, 82).

An effective antiviral response appears to be in place in P-oysters compared to oysters A (**Figure 5**). This response implies the modulation of proteins involved in RIG-I and interferon pathways. An interferon-induced helicase C domain-containing protein, a characteristic domain of RIG-like receptors (RLRs), was only detected in the more resistant Pacific oysters sampled at 26 and 144 hpi. Working as RNA and DNA sensors, RLRs are considered as fundamental receptors of viruses in oysters (75, 83). Several studies have demonstrated that RLR-related genes are highly expressed in Pacific oysters infected with OsHV-1 or poly I:C (39, 84). By sensing nucleic acids of viruses, RLRs strongly contribute to the antiviral defense through the activation of specific signaling pathways, such as the interferon-like pathway (75, 85). In addition, many IFI44-like isoforms, specific of the interferon-like pathway, were modulated in the more resistant Pacific oysters infected with OsHV-1 at 12 and 144 hpi. In vertebrates, the IFN system is a major antiviral response, leading to the transcription of many interferon-stimulated genes (ISGs) that work together to inhibit the replication and spread of viruses (86). Several studies assumed that bivalves, including *C. gigas*, have an IFN-like system equivalent to the vertebrate type I IFN pathway (47, 87, 88). The modulation of IFN-genes, such as IFI44, in *C. gigas* infected with OsHV-1 was first observed by Renault et al. (21) and further experimental infections indicated that the IFN-like response of *C. gigas* limits the replication and spread of OsHV-1 (89). Moreover, studies suggested that IFN genes such as IFI44 play a pivotal role in the resistance of Pacific oysters to OsHV-1 infection. For example, Lafont et al. (90) observed the activation of IFN-related genes in Pacific oysters immune primed with a viral mimic (poly I:C), conferring a better resistance to a second viral infection. Segarra et al. (33) also observed the induction of the IFI44 gene in infected oysters belonging to the family P. Our proteomic results thus confirm the modulation of the IFN pathway in these virus-resistant oysters and underlines once again its significant contribution to the antiviral defense of Pacific oysters.

Numerous proteins which may prevent the proliferation of OsHV-1 were modulated throughout the experimental infection of P-oysters. At the onset of the viral infection (12 hpi), proteins involved in the mRNA surveillance, e.g., decapping enzymes, and proteins involved in polyamine catabolic processes were modulated (**Figure 5**). Several stresses, including viral infection, cause a deregulation of cellular mRNA surveillance. Viruses hijack the host translation machinery to create a cellular environment suitable for their translation and replication (91). Conversely, proteins involved in mRNA degradation, including decapping enzymes and exonucleases, can be used to restrict viral infection (92). Several diamine acetyltransferases (SSATs) involved in polyamine catabolic processes were positively modulated in infected P-oysters sampled at 12 hpi. Viruses utilize polyamines for their transcription and translation (93–95). Therefore, by controlling polyamine levels in cells, diamine acetyltransferases act as viral restriction factors that limit virus proliferation (94, 95).

RNA interference mechanisms also appear implemented in the more resistant Pacific oysters, especially at 144 hpi. In insects, plants and some marine invertebrates, RNAi pathway is considered as a major antiviral defense mechanism by targeting and degrading viral RNA to limit the infection (96, 97). The components of the RNA interference pathway is likely to be present in the Pacific oysters, since several RNAi-related genes were identified (75). However, the contribution of RNAi to the antiviral response of Pacific oysters is not well established. In the present study, a serrate RNA effector was modulated in the more resistant Pacific oysters infected with OsHV-1 at 12 and 144 hpi. Poorly investigated in bivalves, this protein is considered as a key antiviral effector involved in RNA-mediated gene silencing in Drosophila (98). Indeed, this protein has been shown to regulate small interfering RNAs (si- and mi-RNA), which target and degrade viral RNA (98). Enrichment of the gene silencing BP and modulation of the argonaute-2 (AGO-2) support the hypothesis that RNAi mechanisms are involved in the antiviral defense of the more resistant Pacific oysters. By associating with small interfering RNAs and conferring gene silencing, the nuclease AGO-2 was considered as a key protein of the RNAi pathway of bivalves and other invertebrate species (99, 100). Involvement of RNAi in the antiviral defense of Pacific oysters have been hypothesized by some studies (39, 87). Using transcriptomics, He et al. (39) observed an up-expression of the gene DICER in Pacific oysters infected with OsHV-1, which encodes a key protein involved in RNAi mediated antiviral immunity. However, no modulation of other components of the RNAi system could be observed.

A laccase-type phenoloxidase (PO) enzyme was exclusively modulated in infected P-oysters sampled at 26 hpi. In bivalves, PO activities are mainly investigated for their role in the immune defense (101). Indeed, bivalve POs has antimicrobial activity against a broad range of microorganisms, including viruses (101). An increase of PO activity was evident in the scallop *Chlamys farreri* challenged with the acute virus necrobiotic virus (102). Using transcriptomics, two studies reported an upregulation of laccase-related genes in Pacific oysters infected with OsHV-1 (21, 32). Both studies considered the laccase proteins as potential antiviral effectors in *C. gigas*. Interestingly, a cavortin was modulated exclusively in P-oysters sampled at 12 hpi. This protein is the major component of the plasma of *C. gigas* and has been shown to exert antiviral activity against the herpes simplex virus type 1 (HSV-1) (52).

In contrast with the more susceptible Pacific oysters, the more resistant ones produced a suitable molecular response to counteract OsHV-1. Indeed, results suggested that individuals belonging to the P- family present a more rapid and efficient antiviral response compared to Pacific oyster belonging to the more susceptible A-family. This early antiviral response of the more resistant Pacific oysters may quickly block OsHV-1 replication. This proteomic analysis suggests that crosstalk between the IFN-like and RNAi response may occur in *C. gigas* to fight OsHV-1 infection.

## Conclusion

The present proteomic analysis focusing on the host response was consistent with previous transcriptomic analyses performed on Pacific oysters infected with OsHV-1. In addition to validate observations made with transcriptomics approaches, shotgun proteomics highlighted new mechanisms involved in the interactions between *C. gigas* and OsHV-1. This study shows the complementarity of omics approaches, but careful design of the experiment should be proposed to analyze the same samples. This study highlights the benefit of comparative multi-omics performed with groups of animals with different sensitivities to viral infection, and thus could be a landmark example for other subjects.

Protein responses observed in Pacific oysters highly susceptible to the viral infection suggested that the virus is capable of manipulating the immune response of *C. gigas*. The more resistant Pacific oysters develop an effective antiviral response using interferon-like and RNAi pathways. From these results, it is clear that viral responses need to be further studied to better understand how the virus is able to hijack the antiviral response in the more susceptible Pacific oysters using integrated multi-omics, such as proteogenomic approaches. In addition to such discovery oriented methodologies, targeted proteomics would allow monitoring more precisely the dynamics of antiviral proteins. This approach could validate several key proteins involved in host response and define specific biomarkers that could be used for following infection progression and environmental biomonitoring.

## Data Availability Statement

The datasets presented in this study can be found in online repositories. The names of the repository/repositories and accession number(s) can be found in the article/**Supplementary Material**.

## Authors Contributions

This study is the result of a collective work. AS, NF, BM, and TR conceived this study and participated in its design. NF performed the sample preparation for MS/MS analyses. ML, SC, and BM performed proteomic analyses. ML, BM, NF, LD, AS, MP-L, JA, and TR interpreted the results. ML and BM drafted the manuscript. All authors contributed to the article and approved the submitted version.

## Funding

This work received financial support from the European project VIVALDI (H2020 n°678589) and the GESIPHAGIE project supported by the Ifremer scientific direction.

## Conflict of Interest

The authors declare that the research was conducted in the absence of any commercial or financial relationships that could be construed as a potential conflict of interest.
